# Predictive value of NLR combined with CAR in patients with cerebral hemorrhage undergoing maintenance hemodialysis

**DOI:** 10.3389/fneur.2026.1821169

**Published:** 2026-05-18

**Authors:** Yuqing Wang, Yiran Ge, Xiaojie He, Yaqing Wang, Xiaodong Li

**Affiliations:** 1 Graduate School of Hebei Medical University, Shijiazhuang, Hebei, China; 2 Graduate School of Chengde Medical University, Chengde, Hebei, China; 3 Department of Nephrology, Baoding No. 1 Central Hospital of Hebei Medical University, Baoding, Hebei, China

**Keywords:** biomarkers, cerebral hemorrhage, C-reactive protein, neutrophil-to-lymphocyte ratio, renal dialysis

## Abstract

**Objective:**

To assess the predictive value of the neutrophil-to-lymphocyte ratio (NLR), the C-reactive protein-to-albumin ratio (CAR), and their combined use for intracerebral hemorrhage (ICH) in patients undergoing maintenance hemodialysis (MHD), and to assess whether the combined indicator offers incremental discriminative ability beyond either marker alone.

**Methods:**

A total of 335 patients with end-stage renal disease receiving MHD at our hospital between January 2021 and December 2025 were retrospectively enrolled. Based on the occurrence of ICH, patients were divided into an ICH group (*n* = 103) and a non-ICH group (*n* = 232). Demographic and laboratory data were collected to calculate NLR and CAR. Baseline characteristics were compared between groups. Univariate and multivariate logistic regression analyses were conducted to identify independent risk factors for ICH. Receiver operating characteristic curves (ROC) were plotted to assess the discriminative ability of NLR, CAR, and their combination, with the area under the curve (AUC) compared by DeLong's test. Restricted cubic spline modeling was used to examine potential nonlinear relationships, and subgroup analyses were conducted to verify the robustness of the associations.

**Results:**

NLR and CAR levels were significantly higher in the ICH group than in the non-ICH group (*P* < 0.001). After adjusting for confounders including hypertension, multivariate logistic regression identified NLR (OR = 1.24, 95% CI: 1.01–1.54, *P* = 0.048) and CAR (analyzed per standard-deviation increase, OR = 2.44, 95% CI: 1.75–3.41, *P* < 0.001) remained independent factors associated with ICH in MHD patients. ROC analysis yielded AUCs of 0.76 for NLR, 0.80 for CAR, and 0.82 for the combined model. DeLong's test confirmed that the combined indicator outperformed either single marker (*P* < 0.05). RCS analysis suggested a nonlinear positive association of NLR and CAR with ICH risk. Subgroup analyses demonstrated that these associations persisted consistently across different clinical characteristics and dialysis vintages.

**Conclusion:**

NLR and CAR are independent risk markers for ICH in MHD patients, and their combination significantly improves predictive accuracy. This simple, cost-effective inflammation-nutrition composite indicator may serve as a practical tool for the early identification of dialysis patients at high risk of ICH.

## 1 Introduction

Chronic Kidney Disease (CKD) is a global public health concern, defined as persistent kidney structural or functional abnormalities lasting over 3 months, and associated with long-term health consequences. CKD is staged based on estimated glomerular filtration rate (eGFR), with End-Stage Renal Disease (ESRD; eGFR < 15 mL/min/1.73 m^2^) being the final stage, typically necessitating lifelong renal replacement therapy (1). Maintenance Hemodialysis (MHD) is currently one of the most prevalent treatment modalities for ESRD. However, MHD patients face a range of severe complications associated with uremia itself and dialysis treatment, significantly increasing their morbidity and mortality rates (2).

Intracerebral hemorrhage (ICH) is a devastating cerebrovascular event characterized by bleeding within the brain parenchyma due to non-traumatic vascular rupture (3). In the general population, hypertensive arteriolosclerosis is the primary etiology. In MHD patients, who often present with multiple hypertensive complications, the risk of ICH is significantly increased, a phenomenon potentially associated with multiple complex factors. First, the repeated rapid changes in volume and solutes during dialysis lead to significant blood pressure fluctuations and hemodynamic instability, which may induce vascular rupture (4). For another, long-term dialysis patients often present with secondary hyperparathyroidism and calcium-phosphorus metabolic disorders, which leading to vascular calcification and increased vascular fragility (5, 6). Furthermore, the chronic inflammatory state, malnutrition, and underlying cerebrovascular amyloidosis commonly observed in MHD patients collectively form the unique and high-risk pathophysiological basis of ICH (6, 7). The occurrence of ICH in MHD patients is often more severe and has a poorer prognosis, making early identification of high-risk individuals and preventive measures crucial.

In recent years, the role of systemic inflammation in the progression of CKD and cardiovascular complications has garnered increasing attention. The neutrophil-to-lymphocyte ratio (NLR), as a readily available and cost-effective systemic inflammatory marker, effectively reflects the body's inflammatory stress state by integrating information from two key immune cells (neutrophils representing innate immune activation and lymphocytes representing immune regulation). Studies have demonstrated that elevated NLR is independently associated with cardiovascular events, infection risk, and all-cause mortality in patients with ESRD. Similarly, in patients with acute stroke (including ischemic and hemorrhagic types), a higher NLR at admission has been demonstrated to be a strong predictor of neurological deterioration, hematoma enlargement, and poor prognosis (8–10).

The C-reactive protein to albumin ratio (CAR) is another emerging composite biomarker. It combines acute-phase protein C-reactive protein (CRP, representing inflammatory levels) with the nutritional and synthetic function marker albumin (ALB, representing nutritional status, and negative regulation of inflammation), thereby simultaneously assessing both inflammation and nutritional status. This combination is considered to provide a more comprehensive reflection of disease severity and physiological reserves compared to the use of CRP or ALB alone. CAR has been demonstrated to have significant prognostic value in sepsis, heart failure, various malignant tumors, and cardiovascular diseases (11, 12).

Although evidence suggests that both NLR and CAR are valid predictors of overall prognosis in MHD patients, and their combination may provide stronger predictive power (13), current research remains limited on the relationship between these composite markers and the risk of specific fatal complications in MHD patients, such as ICH. Given the potential central role of systemic inflammation and malnutrition in the pathophysiology of ICH in MHD patients, exploring the predictive value of NLR and CAR for this severe event holds significant clinical importance.

Therefore, this study aims to systematically investigate the association between baseline NLR, CAR levels, and their combined indicators with the risk of new-onset ICH in MHD patients through an observational study, and to evaluate the predictive efficacy of these indicators for ICH occurrence. We hypothesize that elevated NLR and CAR levels independently contribute to increased ICH risk in MHD patients, and their combination may provide superior predictive value. The findings are expected to offer a simple and practical tool for early clinical identification of high-risk MHD patients for ICH, thereby providing a basis for implementing individualized monitoring and intervention strategies, ultimately improving patient outcomes.

## 2 Material and methods

### 2.1 Study population

In this retrospective study, we selected clinical data from patients with end-stage renal disease undergoing MHD, who were hospitalized in the Department of Nephrology at Baoding First Central Hospital between January 2021 and December 2025. The study included 164 males and 171 females, aged between 18 and 85 years, with a mean age of approximately 59 years.

The inclusion criteria are as follows: (1) Inpatients aged ≥17 years, with no gender restriction. (2) MHD duration ≥3 months, the dialysis frequency is 2–3 times a week. (3) The study participants were MHD patients who were hospitalized during the same period and underwent head CT examinations. Among them, patients with head CT-confirmed ICH occurring during the dialysis period were assigned to the cases group, while for the controls group, CT was performed due to various clinical indications during the same hospitalization period, including evaluation of non-specific neurological symptoms (e.g., dizziness, headache, or transient weakness not attributable to acute stroke), routine pre-operative assessment for vascular access surgery, or exclusion of intracranial pathology in the context of hypertensive urgency. The absence of acute ICH in control participants was confirmed by formal radiology reports documenting no evidence of acute intracranial hemorrhage.

**Figure 1:**
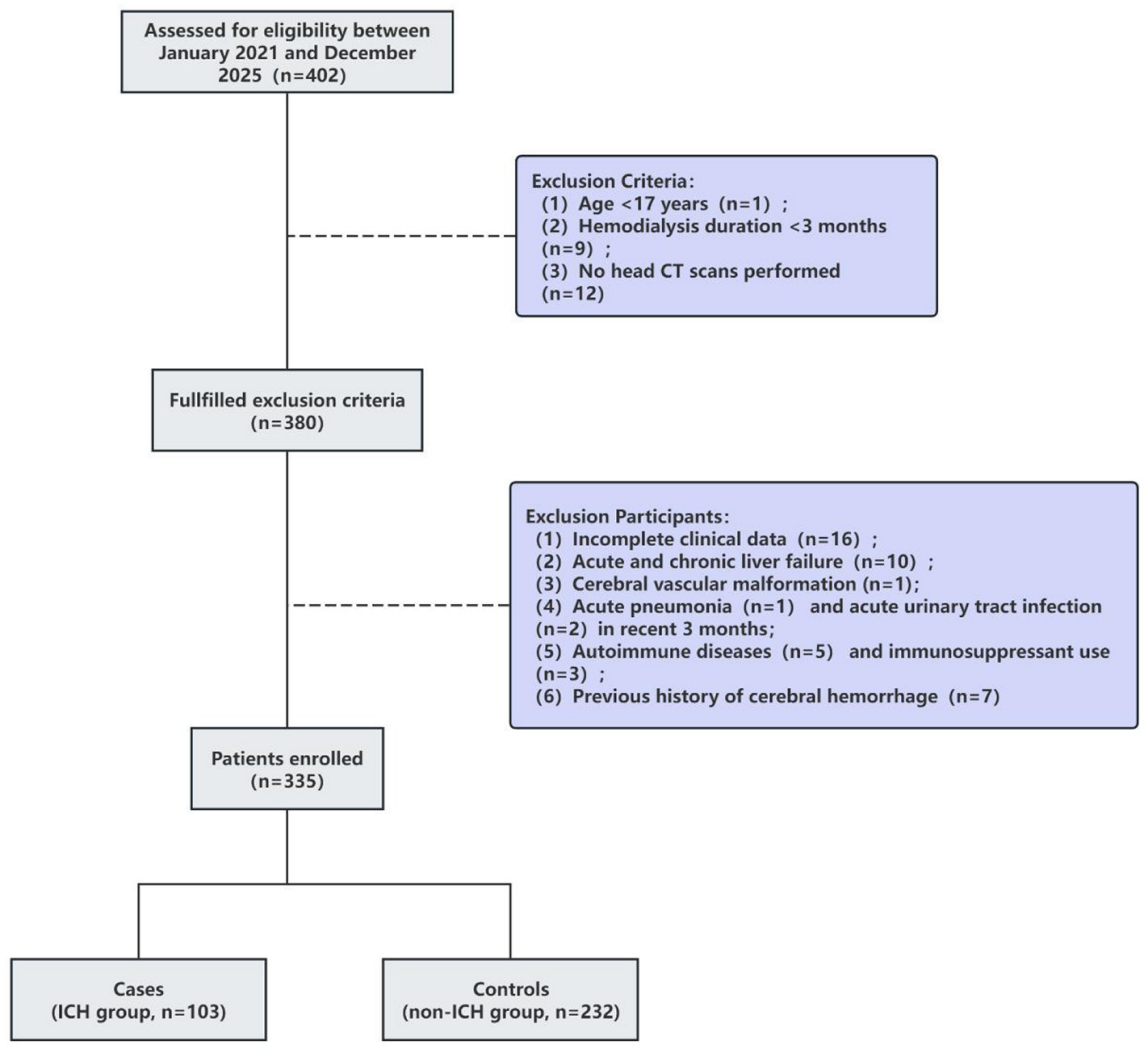
Flowchart of participants selection. ICH, intracerebral hemorrhage.

The exclusion criteria are as follows: (1) Missing experimental data or other incomplete clinical information. (2) Presence of chronic or acute liver failure. (3) History of traumatic brain injury, aneurysm, vascular malformation, Moyamoya disease, malignancy, or contraindications to anticoagulants or antiplatelet agents. (4) History of major surgery, severe trauma, or acute inflammation. (5) Presence of autoimmune diseases or current use of immunosuppressive therapy. (6) History of intracerebral hemorrhage. Figure 1 illustrates the participant selection process, with 335 patients screened and 103 included in the ICH group.

### 2.2 Research methods

#### 2.2.1 Data collection

All patients were treated with regular hemodialysis, 2–3 times per week for 4 h each time, using an autogenous arteriovenous fistula as vascular access for dialysis. Dialysis parameters were as follows: blood flow 200–250 mL/min, dialysate flow 500 mL/min, standard bicarbonate dialysate was used, and dialysate calcium concentration was 1.25 mmol/L. The anticoagulation regimen was low-molecular-weight heparin (low-molecular-weight heparin sodium or low-molecular-weight heparin calcium), with the dose adjusted according to the patient's body weight.

Collect demographic data and laboratory test results of the enrolled subjects, including age, gender, smoking history, alcohol consumption history, history of hypertension, frequency of hemodialysis, duration of hemodialysis, history of diabetes mellitus, hemoglobin (HB), white blood cell count (WBC), neutrophil count (NE), lymphocyte count (LYM), platelet count (PLT), monocyte count (MONO), fasting plasma glucose (FPG), C-reactive protein (CRP), albumin (ALB), serum creatinine (SCR), serum uric acid (SUA), total cholesterol (TC), low-density lipoprotein cholesterol (LDL-C), parathyroid hormone (PTH), serum calcium (Ca), and serum phosphorus (P). The laboratory indices analyzed in this study (including NLR and CAR) were obtained from routine pre-dialysis follow-up records within 30 days prior to the ICH event for the ICH group, and from the most recent routine dialysis follow-up during the study period for the non-ICH group. No blood samples were collected after ICH onset. This temporal sequence establishes a clear antecedent-exposure-to-outcome relationship, supporting the interpretation of NLR and CAR as baseline predictors rather than acute-phase reactants.

#### 2.2.2 NLR, CAR hematological parameters and calculation formulas

Calculate NLR and CAR: NLR = (NE counts ( × 10^9^/L) / LY counts ( × 10^9^/L)) (14), CAR = CRP (mg/L) / ALB (g/L) (15).

#### 2.2.3 Assessment of intracerebral hemorrhage

All patients underwent cranial CT scans by radiologists upon admission, with diagnostic criteria including: (1) acute onset of neurological symptoms; (2) CT imaging demonstrating well-defined intracerebral hyperdense hemorrhagic lesions. The imaging evaluation specifically included: hemorrhage location, hematoma volume (calculated using the Takeda formula), hematoma morphology, presence of ventricular rupture, and mass effect (16, 17). Radiologists were responsible for drafting the imaging reports.

### 2.3 Statistical analysis

Statistical analysis was performed using SPSS 27.0 and R language 4.1.3 software. Measurement data conforming to normal distribution were expressed as mean ± standard deviation (x ± s), with intergroup comparisons conducted using the independent samples *t*-test. Non-normally distributed data were presented as median, and intergroup comparisons were performed using the Mann-Whitney U test. Categorical data were expressed as frequency [percentage, *n* (%)], with intergroup comparisons using the χ^2^ test. First, the differences in baseline characteristics between the ICH group and the non-ICH group were compared using the aforementioned testing methods. Variables with *P* < 0.05 in univariate analysis and clinically significant variables were included in the multivariate logistic regression model to control for confounding factors and screen for independent predictors associated with ICH. Variables were selected for the multivariate model based on a combination of statistical significance in univariate analysis (*P* < 0.05) and clinical relevance established by prior literature (e.g., age, sex, and history of hypertension). All candidate variables were entered simultaneously using the forced entry method rather than stepwise selection to minimize the risk of overfitting. Multicollinearity was assessed using the variance inflation factor (VIF), with all variables in the final model showing VIF < 5, indicating no significant collinearity. With 103 events and 5 predictors in the final model, the events-per-variable ratio was approximately 20:1, exceeding the commonly recommended minimum of 10:1, supporting the stability of the model. Results were expressed as odds ratio (OR) and its 95% confidence interval (CI). To evaluate the discriminative ability of NLR, CAR, and their combined indices for ICH, receiver operating characteristic (ROC) curves were plotted, and the area under the curve (AUC) was calculated. The combined indicator was derived from a multivariable logistic regression model incorporating both NLR and CAR as continuous predictors. The predicted probabilities from this model were used to generate the combined ROC curve. The model specification was: Logit(P) = β0 + β1 × NLR + β_2_ × CAR. The DeLong test was used to compare AUCs pairwise to determine whether the combined indices had incremental predictive value. To further investigate the association patterns between risk factors and indicator levels, NLR and CAR were stratified by the three-tail method for trend analysis, and multiple models were constructed for validation: Model 1, unadjusted status; Model 2, adjusted for age and sex as covariates; Model 3, which introduced variables from Model 2 to further adjust for various potential confounding factors related to clinical outcomes of ICH and refined the indicators. Simultaneously, a restricted cubic spline (RCS) model was employed to fit the nonlinear relationship between NLR, CAR, and the log-odds ratio (Log Odds) of ICH risk after confounding factors adjustment. Finally, stratified logistic regression models were used for subgroup analysis (stratified by sex, hypertension, diabetes, smoking history, alcohol consumption history, and dialysis duration), and forest plots were drawn to test the robustness of the association between NLR and CAR with ICH and its consistency across different populations. Interactions were evaluated using the likelihood ratio test. A significance level of p < 0.05 was considered statistically significant.

## 3 Results

### 3.1 Baseline characteristics of study participants and univariate, multivariate logistic regression analysis

The study ultimately included 335 MHD patients, with 103 patients in the ICH group and 232 patients in the non-ICH group. Given the continuous nature of the CAR variable, to facilitate analysis, CAR values were standardized (CAR_SD), with each standard deviation (SD) serving as a unit of analysis (1 SD = 0.24). This method has been widely used in the risk quantification of biomarkers (18). The Z-score transformation was applied to facilitate interpretation of the odds ratio as the change in ICH risk per one standard deviation increase, providing a clinically meaningful unit of comparison independent of the original measurement scale. CAR_SD was used exclusively for logistic regression analyses and trend analyses, whereas the original CAR values were retained for ROC analysis to preserve clinically interpretable cut-off values. Univariate logistic regression analysis revealed that a history of hypertension, dialysis duration ≥ 36 months, WBC, MONO, NLR, and CAR_SD were significantly associated with the risk of ICH in MHD patients (*P* < 0.05). Specifically, patients with a history of hypertension had a 2.40-fold increased risk of ICH compared to those without hypertension (OR = 2.40, 95%CI:1.31–4.38).After adjusting for the variables with *P* < 0.05 in the univariate analysis, as well as clinically relevant factors (such as age, gender, etc.), in a multivariate logistic regression model, the results indicated that a history of hypertension, NLR, and CAR remained independent risk factors for ICH in MHD patients (Table 1). In particular, the adjusted OR for NLR was 1.24 (95%CI:1.01–1.54, *P* = 0.048), and the adjusted OR for CAR_SD was 2.44 (95%CI:1.75–3.41, *P* < 0.001), confirming that both NLR and CAR have independent predictive value for ICH even after controlling for traditional risk factors.

**Table 1:** Univariate and multivariate logistic regression analyses for ICH in MHD patients.

Variables	Univariate analysis		Multivariate analysis	
	*P*	OR (95%CI)	*P*	OR (95%CI)
Sex, *n* (%)	0.920	1.02 (0.64 ~ 1.63)		
Hypertension, *n* (%)	**0.004**	2.40 (1.31 ~ 4.38)	**0.010**	2.65 (1.26 ~ 5.54)
Diabetes, *n* (%)	0.252	0.76 (0.48 ~ 1.21)		
Smoking status, *n* (%)	0.160	1.41 (0.87 ~ 2.29)		
Alcohol consumption, *n* (%)	0.636	0.87 (0.48 ~ 1.57)		
MHD frequency				
2 times/week		1.00 (Reference)		
3 times/week	0.061	0.42 (0.17 ~ 1.04)		
MHD duration (years)				
< 3		1.00 (Reference)		
3–5	0.216	1.42 (0.82 ~ 2.47)		
≥5	0.002	3.82 (1.62 ~ 9.04)	**0.004**	5.02 (1.70 ~ 14.84)
Age (years)	0.652	1.00 (0.99 ~ 1.02)		
HB(g/L)	0.414	1.00 (0.99 ~ 1.02)		
WBC ( × 10^9^/L)	**0.031**	1.09 (1.01 ~ 1.19)	0.058	1.11 (1.00 ~ 1.24)
NLR	**< 0.001**	1.51 (1.29 ~ 1.77)	**0.048**	1.24 (1.01 ~ 1.54)
MONO ( × 10^9^/L)	**0.038**	2.70 (1.06 ~ 6.92)	0.303	2.10 (0.51 ~ 8.67)
PLT ( × 10^9^/L)	0.615	1.00 (1.00 ~ 1.00)		
FPG (mmol/L)	0.688	1.02 (0.92 ~ 1.13)		
CAR^*^ (perSD)	**< 0.001**	2.59 (2.00 ~ 3.36)	**< 0.001**	2.44 (1.75 ~ 3.41)
SCR (μmol/L)	0.060	1.00 (1.00 ~ 1.00)		
SUA (μmol/L)	0.281	1.00 (1.00 ~ 1.00)		
TC (mmol/L)	0.274	1.09 (0.94 ~ 1.26)		
LDL-C (mmol/L)	0.576	1.06 (0.85 ~ 1.33)		
PTH (pg/mL)	0.150	1.00 (1.00 ~ 1.00)		
Ca (mmol/L)	0.768	0.89 (0.40 ~ 1.95)		
P (mmol/L)	0.900	1.02 (0.71 ~ 1.47)		

^*^CAR variable was standardized (Z-score transformation) for ease of interpretation, with a mean of 0 and a standard deviation (SD) of 0.24. The OR reported corresponds to the change in ICH risk per one SD increase in CAR

ICH, intracerebral hemorrhage; MHD, maintenance hemodialysis; HB, hemoglobin; WBC, white blood cell count; MONO, monocyte count; PLT, platelet count; FPG, fasting glucose; SCR, serum creatinine; SUA, serum uric acid; TC, total cholesterol; LDL-C, low-density lipoprotein cholesterol; PTH, parathyroid hormone; Ca, serum calcium; P, serum phosphorus.

Bold P values indicate statistical significance (P < 0.05).

### 3.2 ROC curve analysis of NLR, CAR, and combined metrics

**Figure 2:**
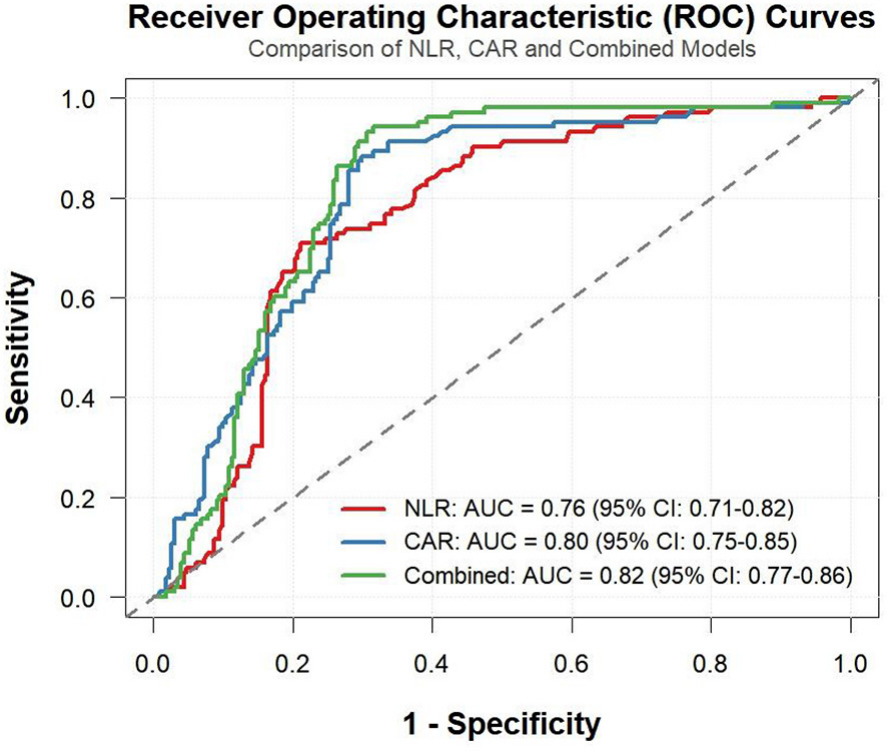
ROC curves of NLR, CAR, and their combination for predicting ICH in MHD patients. NLR, neutrophil-to-lymphocyte ratio; CAR, C-reactive protein to albumin ratio; ICH, intracerebral hemorrhage; MHD, maintenance hemodialysis.

ROC curve analysis revealed that NLR, CAR, and their combined indicator all exhibited good discriminative ability for ICH in MHD patients (Figure 2). Among the individual indicators, CAR showed slightly better discriminative ability than NLR: the AUC for CAR was 0.80 (95% CI: 0.75–0.85), with a sensitivity of 70% (95% CI: 0.64–0.76) and specificity of 88% (95% CI: 0.82–0.95) when the cutoff value was 0.234. The positive predictive value (PPV) was 93% (95% CI: 0.89–0.97), and the negative predictive value (NPV) was 57% (95% CI: 0.49–0.65). For NLR, the AUC was 0.76 (95%CI: 0.71–0.82), with a sensitivity of 79% (95%CI: 0.74–0.84) and specificity of 71% (95% CI: 0.62–0.80) when the cutoff value was 2.92. The PPV was 86% (95% CI: 0.81-0.91), and the NPV was 60% (95% CI: 0.51–0.69). The combined predictive model showed the best discriminative ability, with the AUC increasing to 0.82 (95% CI: 0.77–0.86). The curve was closer to the upper-left corner of the ROC plot, indicating that the combined indicator can more accurately distinguish between ICH and non-ICH patients (Table 2).

**Table 2:** Predictive value of NLR and CAR for ICH risk.

Indicator	AUC (95%CI)	Accuracy (95%CI)	Sensitivity (95%CI)	Specificity (95%CI)	PPV (95%CI)	NPV (95%CI)	Cut off
NLR	0.76 (0.71–0.82)	0.76 (0.72–0.81)	0.79 (0.74–0.84)	0.71 (0.62–0.80)	0.86 (0.81–0.91)	0.60 (0.51–0.69)	2.92
CAR	0.80 (0.75–0.85)	0.76 (0.71–0.80)	0.70 (0.64–0.76)	0.88 (0.82–0.95)	0.93 (0.89–0.97)	0.57 (0.49–0.65)	0.234

AUC, area under curve; NLR, neutrophil-to-lymphocyte ratio; CAR, C-reactive protein to albumin ratio; ICH, intracerebral hemorrhage; MHD, maintenance hemodialysis.

### 3.3 DeLong test results

The DeLong test was performed to compare the differences in AUC values among various predictive indicators. The results (Table 3) showed that the AUC difference between the combined indicator and NLR was 0.0632, and the AUC difference between the combined indicator and CAR was 0.0198, both of which were statistically significant. This indicates that the predictive performance of the combined indicator was significantly superior to that of either NLR or CAR alone. However, the AUC difference between NLR and CAR was not statistically significant, suggesting that the predictive capabilities of the two indicators at the single-level were comparable.

**Table 3:** DeLong test comparing differences in AUC among different predictive indicators.

Predictive index	Comparison of the AUCs	Standard error	95%CI	*Z* values	*P* values
Combined vs. NLR	0.0632	0.0244	0.0153 ~ 0.1111	2.5873	0.0097
Combined vs. CAR	0.0198	0.0094	0.0014 ~0.0382	2.1069	0.0351
NLR vs. CAR	−0.0434	0.0303	−0.1028 ~0.0160	−1.4333	0.1518

AUC, area under curve; NLR, neutrophil-to-lymphocyte ratio; CAR, C-reactive protein to albumin ratio.

### 3.4 Trend analysis results

NLR and CAR were divided into tertiles for trend analysis, and multi-model adjustments were conducted to validate the dose-response relationships. The results showed that, regardless of whether confounding factors were adjusted for, the risk of ICH in MHD patients increased significantly with higher levels of NLR and CAR (all *P* < 0.001). For NLR (Table 4): In the unadjusted model (Model 1), compared with the lowest tertile group (NLR < 1.97), the OR for the middle tertile group (1.97 ≤ NLR ≤ 3.07) was 2.69 (95% CI:1.26–5.76, *P* = 0.011), and for the highest tertile group (NLR > 3.07), the OR was 12.91 (95% CI: 6.24–26.74, *P* < 0.001). After adjusting for age and sex (Model 2), and further adjusting for hypertension, WBC, MONO, and CAR (Model 3), the OR for the highest tertile group remained as high as 8.43 (95% CI: 3.66–19.41, *P* < 0.001), indicating a robust increasing trend. For CAR (Table 5): In Model 1, compared with the lowest tertile group (CAR < 0.17), the OR for the middle tertile group (0.17 ≤ CAR ≤ 0.43) was 8.18 (95% CI: 3.28–20.39, *P* < 0.001), and for the highest tertile group (CAR > 0.43), the OR reached 21.35 (95% CI: 8.65–52.70, *P* < 0.001). After multivariable adjustment (Model 3), the OR for the highest tertile group remained significant at 15.23 (95% CI: 5.89–39.43, *P* < 0.001), confirming a strong dose-response relationship.

**Table 4:** Trend analysis and multi-model adjustment of NLR tertiles with ICH risk.

Variables	Model 1		Model 2		Model 3	
	OR (95%CI)	*P*	OR (95%CI)	*P*	OR (95%CI)	*P*
NLR < 1.97	1.00 (Reference)		1.00 (Reference)		1.00 (Reference)	
1.97 ≦ NLR ≦ 3.07	2.69 (1.26 ~ 5.76)	**0.011**	2.85(1.32~ 6.14)	**0.007**	2.69(1.20 ~ 6.02)	**0.016**
NLR > 3.07	12.91(6.24 ~ 26.74)	**< 0.001**	14.05(6.69~29.48)	**< 0.001**	8.43(3.66 ~ 19.41)	**< 0.001**

Model 1: unadjusted.

Model 2: model 1+ age and sex.

Model 3: model 2+ hypertension, WBC, MONO, and CAR.

*P* for trend < 0.001 across all models.

NLR, neutrophil-to-lymphocyte ratio; ICH. intracerebral hemorrhage; WBC, white blood cell count; MONO, monocyte count; CAR, C-reactive protein to albumin ratio.

Bold P values indicate statistical significance (P < 0.05).

**Table 5:** Trend analysis and multi-model adjustment of CAR tertiles with ICH risk.

Variables	Model 1		Model 2		Model 3	
	OR (95%CI)	*P*	OR (95%CI)	*P*	OR (95%CI)	*P*
CAR < 0.17	1.00 (Reference)		1.00 (Reference)		1.00 (Reference)	
0.17 ≦ CAR ≦ 0.43	8.18 (3.28 ~ 20.39)	**< 0.001**	8.24(3.30 ~ 20.54)	**< 0.001**	7.21 (2.85 ~ 18.24)	**< 0.001**
CAR > 0.43	21.35 (8.65~52.70)	**< 0.001**	21.81(8.81~53.99)	**< 0.001**	15.23 (5.89 ~ 39.43)	**< 0.001**

Model 1: unadjusted.

Model 2: model 1+ age and sex.

Model 3: model 2+ hypertension, WBC, MONO, and NLR.

*P* for trend < 0.001 across all models.

CAR, C-reactive protein to albumin ratio; ICH, intracerebral hemorrhage; WBC, white blood cell count; MONO, monocyte count; NLR, neutrophil-to-lymphocyte ratio.

Bold P values indicate statistical significance (P < 0.05).

### 3.5 Analysis results of restricted cubic spline (RCS)

**Figure 3:**
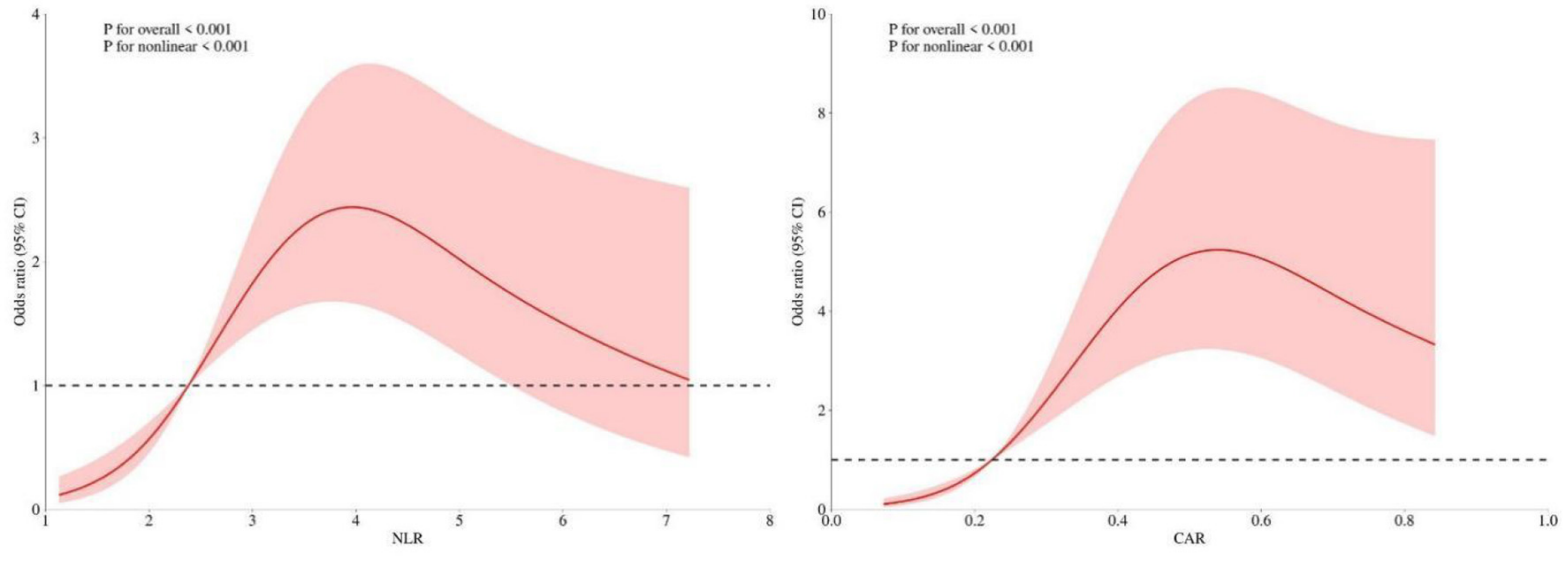
RCSs analysis of the association between NLR, CAR, and ICH risk. NLR, neutrophil-to-lymphocyte ratio; CAR, C-reactive protein to albumin ratio; ICH, intracerebral hemorrhage.

The RCS model was used to fit the relationship between NLR, CAR, and the log odds of ICH risk after adjusting for age, sex, hypertension, WBC, MONO, and another indicator (CAR or NLR). The results showed that both NLR and CAR were nonlinearly positively correlated with the risk of ICH (overall *P* < 0.001, nonlinear *P* < 0.001) (Figure 3). The RCS curve for NLR showed that when NLR ≤ 2.38, the ICH risk increased rapidly with rising NLR; however, when NLR > 2.38, the rate of increase slowed down, and in the high-value range, the risk plateaued or slightly decreased. The RCS curve for CAR showed that when CAR ≤ 0.224, the ICH risk began to rise gradually; when CAR > 0.224, the risk curve steepened significantly, indicating a marked increase in risk after this inflection point. In the extremely high CAR range, the risk plateaued and then slightly declined.

These nonlinear relationships suggest that the effects of inflammation and nutritional imbalance on ICH risk may involve threshold effects, rather than a simple linear accumulation. The observed plateau at extreme values should be interpreted cautiously. Several factors may contribute to this pattern, including: (1) limited sample size in the upper tails of the distribution (*n* = 23 for NLR >5; *n* = 18 for CAR >1.0), leading to estimate instability; (2) potential survival bias, as patients with extremely high inflammatory burden may experience competing fatal events before ICH can occur; or (3) true biological threshold effects. Additional studies with larger sample sizes and prospective designs are needed to clarify the shape of this association.

### 3.6 Exploration of subgroup analysis

**Figure 4:**
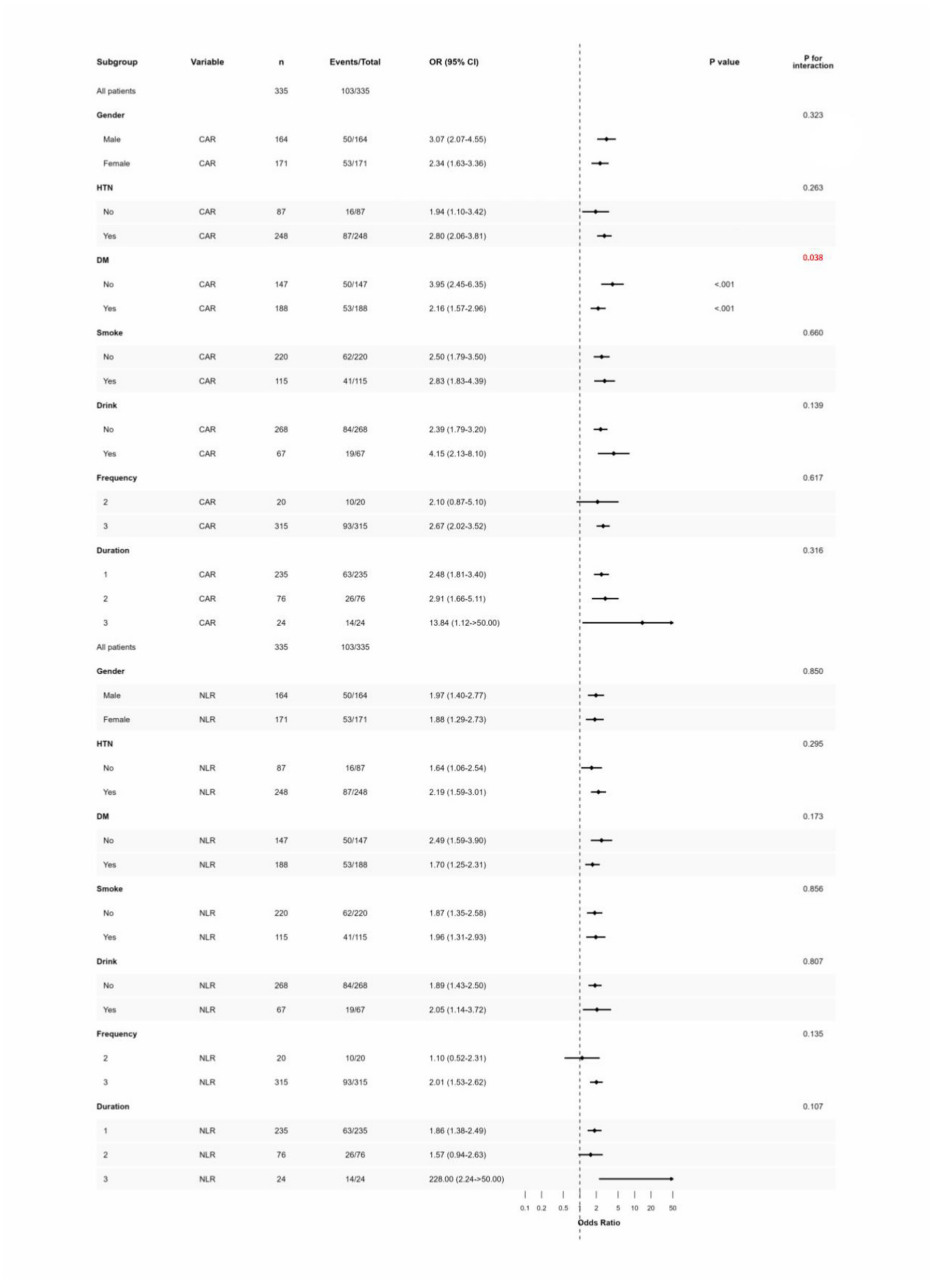
Forest plot of subgroup analyses for NLR and CAR in predicting ICH risk. NLR, neutrophil-to-lymphocyte ratio; CAR, C-reactive protein to albumin ratio; ICH, intracerebral hemorrhage; HTN, hypertension; DM, diabetes. Frequency:2:2 times/week;3:3 times/week. Duration:1: < 3years;2:3~5 years;3:≥5 years.

Subgroup analyses revealed that both NLR and CAR were positively correlated with ICH risk across all subgroups, with the associations remaining robust in different populations (Figure 4). There was a significant difference in the OR of CAR between diabetic and non-diabetic groups, with an interaction *P*-value < 0.001. Moreover, as dialysis duration increased, the OR gradually elevated, especially in the subgroup with dialysis duration ≥ 60 months, where the OR of CAR reached 13.84. NLR showed a higher OR in hypertensive patients, and in long-term dialysis patients, its OR further increased to 228.00, suggesting that prolonged dialysis may significantly enhance cerebrovascular risks related to inflammation and immune imbalance. Apart from the significant interaction of diabetes status with CAR, no significant heterogeneity was found between other subgroups, with all interaction *P*-values > 0.05, supporting the wide applicability of these two indicators as predictive markers.

## 4 Discussion

This study is the first to systematically and simultaneously evaluate both the NLR and the CAR, examining their combined predictive value that has not been previously explored. Addressing this gap, our study is the first to integrate NLR, an indicator reflecting immune cell imbalance, with CAR, a marker representing the inflammation–nutrition composite status, to construct a more comprehensive risk assessment approach. The results demonstrated that both elevated NLR and CAR levels were independently and positively associated with an increased risk of ICH. Furthermore, their combination provided significant incremental discriminative ability, suggesting that the interplay between inflammatory activation and nutritional depletion plays a central role in the pathophysiological process of ICH among MHD patients.

### 4.1 Impact of traditional risk factors and dialysis duration on hypertension and ICH

Before exploring novel inflammatory and nutritional biomarkers, it is essential to recognize that traditional risk factors continue to play a vital role in the pathogenesis of ICH among patients undergoing MHD. The multivariate analysis reaffirms that both hypertension and a dialysis vintage ≥60 months are independent risk factors for ICH in this population, with adjusted OR of 2.65 (95% CI: 1.26–5.54) and 5.02 (95% CI: 1.70–14.84), respectively. These findings underscore the continued significance of these factors in ICH risk stratification. Hypertension remains the most well-established and potent traditional risk factor for ICH in MHD patients. Notably, this study is the first to establish a dialysis duration of ≥60 months as an independent risk factor for ICH in a multivariate analysis. The underlying pathophysiological mechanisms in patients undergoing MHD are complex and multifaceted. First, hypertension is highly prevalent and notoriously difficult to manage in this population due to factors such as sodium and fluid retention, activation of the renin-angiotensin-aldosterone system, and endothelial dysfunction (19). Second, the hemodialysis process itself introduces intermittent volume shifts and solute removal, leading to marked intradialytic and interdialytic blood pressure fluctuations, including episodes of intradialytic hypotension. This hemodynamic “roller coaster” imposes repeated shear stress on already atherosclerotic or lipohyalinotic intracerebral vessels, significantly increasing the risk of vessel rupture (4, 20). Additionally, chronic hypertension and prolonged dialysis duration frequently correlate with secondary hyperparathyroidism and calcium–phosphate metabolism disturbances, which are common in MHD patients. These factors synergistically promote intracerebral vascular calcification and stiffness, further compromising vascular compliance (21, 22). Consequently, while there is a growing focus on inflammatory mechanisms, maintaining strict blood pressure control and implementing individualized dialysis prescriptions to ensure hemodynamic stability—alongside risk stratification based on dialysis duration—are essential strategies for preventing ICH in MHD patients. For patients with a dialysis duration exceeding 5 years, even with well-controlled blood pressure, heightened vigilance is crucial. Such individuals should be regarded as high-risk for ICH, and should undergo regular cerebrovascular assessment sand comprehensive clinical management.

### 4.2 NLR, inflammation-immune imbalance, and cerebrovascular fragility

Patients on MHD are characterized by a state of chronic low-grade inflammation and immune dysregulation, which contribute to endothelial dysfunction, oxidative stress, and blood-brain barrier disruption—key factors that increase the risk of intracerebral hemorrhage ICH (20, 23). Consistent with this pathophysiological background, our findings confirm that an elevated NLR is an independent risk factor for ICH in this population.

As key mediators of innate immunity, neutrophils—when overactivated—release substantial quantities of reactive oxygen species (ROS), matrix metalloproteinases (MMPs, e.g., MMP-9), and neutrophil extracellular traps (NETs). In patients with CKD, impaired clearance of these mediators leads to sustained endothelial injury, resulting in degradation of tight junction proteins (e.g., ZO-1, occludin), increased blood-brain barrier permeability, and ultimately, greater vascular fragility (23, 24). Moreover, lymphopenia—reflected in an elevated NLR—often signifies immune exhaustion and compromised regulatory function, attenuating the negative feedback control of inflammation. This persistent low-grade inflammatory milieu promotes hyaline degeneration and fibrinoid necrosis in small arteries—hallmark pathological features of hypertensive ICH (25, 26). Patients with chronic kidney disease, especially those in the MHD stage, are chronically exposed to low-grade inflammation. NLR, as a key marker of systemic inflammatory response, reflects the balance between neutrophil-mediated pro-inflammatory responses and lymphocyte-mediated immune regulation. Previous studies have shown that an elevated NLR is associated with all-cause mortality, cardiovascular and cerebrovascular events, and adverse outcomes in stroke among MHD patients (27, 28).

Our RCS analysis revealed a nonlinear relationship between NLR and ICH risk, characterized by a steep rise at moderate NLR levels, followed by a plateau at the highest values. This pattern may reflect an immune paralysis state following an “inflammatory storm,” or a competing risk from other complications such as severe infections or sudden cardiac death in patients with extremely high inflammation levels, preventing them from surviving long enough to experience ICH. This phenomenon may be attributable to immune exhaustion or compensatory downregulation of the inflammatory response (29, 30). This complex relationship suggests that NLR is not only an indicator of inflammation levels but also serves as a “barometer” for immune system function. Its role in cerebrovascular damage may have a threshold effect, which requires further validation in prospective studies.

### 4.3 The inflammation–malnutrition vicious cycle: CAR as a superior integrated risk indicator

In this study, CAR showed a stronger predictive value for ICH risk than NLR, suggesting that the combined effect of inflammation and malnutrition may exert a more deleterious influence in MHD patients than inflammation alone. As a composite index, CAR simultaneously reflects the inflammatory burden and nutritional deficiency, effectively capturing the core components of this pathological cycle. The analysis shows that for each 1 SD increase in CAR, the risk of ICH significantly increases, further suggesting that CAR could be an effective risk marker for ICH in patients undergoing MHD. Although the analysis of CAR_SD enhances the interpretability of the results, it is still important to note that individualized assessment should be based on specific value changes in clinical practice. Future studies may further explore the clinical cutoff value of CAR.

CAR is a composite biomarker combining acute-phase inflammatory protein CRP with nutritional marker albumin, which provides a more comprehensive reflection of the dual imbalance in inflammation and nutritional status. Previous studies have demonstrated that CAR outperforms CRP or albumin alone in prognosticating outcomes in various malignancies (31). On one hand, sustained elevation of CRP is an acute-phase response generated by the liver under the influence of pro-inflammatory cytokines such as interleukin-6 (IL-6) and tumor necrosis factor-α (TNF-α). These cytokines directly impair endothelial integrity, suppress hepatic albumin synthesis, and promote systemic protein catabolism, contributing to a state of chronic inflammation and malnutrition (32). On the other hand, dialysis patients are chronically exposed to uremic toxin accumulation, oxidative stress, and inflammation, often leading to protein-energy wasting (PEW) and hypoalbuminemia (33). This “inflammation-malnutrition double blow” state weakens vascular repair capabilities, increases capillary permeability, and raises the risk of small vessel rupture, making CAR a sensitive indicator of ICH risk (33, 34). The study found a strong positive correlation between elevated CAR and the risk of ICH, which remained significant even after adjusting for various potential confounders. Trend analysis showed a sharp rise in ICH risk when CAR ≥ 0.43, providing a clear quantitative threshold for clinical identification of patients at extremely high risk due to the dual impact of inflammation and malnutrition. This suggests that the combination of high inflammation levels and malnutrition isa critical threshold for ICH risk. These findings align with previous evidence supporting the predictive utility of CAR in oncological and cardio-cerebrovascular contexts (35, 36).

### 4.4 Incremental discriminative value and clinical significance of combined indicators

Single biomarkers typically reflect only one aspect of complex pathophysiological processes. Our study highlights that the combination of NLR and CAR provides a synergistic discriminative effect, surpassing the discriminative value of each biomarker individually. The synergistic effect is supported by biological rationale: NLR primarily reflects acute immune-inflammatory responses at the cellular level, while CAR captures the chronic, systemic inflammation and nutritional status. Together, these markers offer a more comprehensive pathophysiological profile, spanning from immune dysregulation and inflammation to nutritional depletion.

Although the combined indicator achieved a statistically significant improvement in AUC compared to CAR alone (ΔAUC = 0.0198, DeLong test *P* = 0.035), the magnitude of this incremental discrimination was modest. From a clinical implementation perspective, this small gain must be weighed against the practical advantage of using a single biomarker vs. a combined index. We recommend that future prospective studies evaluate whether this statistical improvement translates into meaningful changes in clinical decision-making, ideally through decision curve analysis or net reclassification improvement metrics. In settings where both NLR and CAR are routinely available at no additional cost, using both may provide marginal benefit without added burden.

From a translational medicine perspective, the combination of NLR and CAR holds significant potential for clinical application. Firstly, these biomarkers are cost-effective, as they are derived from routine blood tests without incurring additional costs. Secondly, they allow for dynamic monitoring, as both values can be assessed through routine hematological and biochemical tests in dialysis centers, enabling longitudinal risk assessment. The study suggests that MHD patients with CAR > 0.4 and NLR > 3.0 should be considered at very high risk for ICH and managed with intensified interventions, including: 1. Rigorous blood pressure control, with home BP monitoring during interdialytic periods and tailored antihypertensive regimens to minimize hemodynamic fluctuations; 2. Customized anticoagulation management, balancing bleeding and thrombosis risks while exercising caution with anticoagulant use; 3. Active nutritional interventions, such as dietary counseling, oral nutritional supplements, and enteral nutrition support when necessary, to improve albumin levels; and 4. Improved education and follow-up, with emphasis on educating patients and their families about the early warning signs of ICH. Future studies should focus on developing predictive nomograms or risk scores based on NLR and CAR, and compare them with traditional clinical scores (such as the ICH score) to further promote their clinical application. From a clinical perspective, the combined assessment of NLR and CAR may assist clinicians in identifying high-risk populations, allowing for individualized adjustments to dialysis parameters, anticoagulation intensity, blood pressure control, and nutritional interventions, ultimately reducing the risk of intracerebral hemorrhage.

### 4.5 Limitations and future prospects

This study is the first to specifically focus on MHD patients and clearly demonstrates the predictive value of NLR and CAR for ICH risk in this population. It features detailed trend analyses, an exploration of nonlinear associations, and comprehensive multidimensional subgroup analyses, leading to robust conclusions. Nevertheless, several limitations should be acknowledged. First, the retrospective design precludes establishing a definitive causal or temporal relationship between NLR/CAR and ICH, and residual confounding cannot be entirely ruled out. Second, as a single-center study conducted in northern China, the findings may not be directly generalizable to populations with different demographics, dialysis practices, or healthcare systems. Variations in dialysis protocols, anticoagulation strategies, and baseline inflammatory profiles across regions may influence the predictive performance of NLR and CAR. Third, measuring the indicators at only a single time point fails to capture the relationship between their dynamic changes and associated risks. Finally, other factors that may have influenced the results—such as therapeutic medications, dialysis anticoagulation protocols, and dialysis modalities—were not included in the analysis. Fifth, the subgroup analyses, particularly those stratified by dialysis duration, included limited sample sizes in certain strata, which may have contributed to unstable effect estimates and wide confidence intervals. Although these data reflect the actual clinical distribution in our center, the corresponding odds ratios should be interpreted with caution and considered exploratory rather than confirmatory.

Although these data reflect the actual clinical distribution in our center, the corresponding odds ratios should be interpreted with caution and considered exploratory rather than confirmatory. Consequently, the findings of this study should be interpreted as hypothesis-generating and require further validation in prospective cohort studies before they can be translated into clinical risk prediction tools.

Additionally, several potential confounders could not be fully adjusted for due to the retrospective nature of data collection. Detailed information on anticoagulant and antiplatelet medication use was not uniformly available, although procedural anticoagulation with heparin is standard for all MHD patients at our center. Longitudinal blood pressure recordings and antihypertensive medication histories were not collected, though a history of hypertension was included as a covariate. Formal dialysis adequacy metrics such as Kt/V were not consistently documented; however, dialysis frequency and duration were included in the analysis as proxies for dialysis exposure. While these unmeasured factors represent limitations, the consistency and magnitude of the observed associations suggest that residual confounding is unlikely to fully account for the findings.

Future studies should aim to advance in the following areas: First, conduct multicenter prospective cohort studies with dynamic monitoring of NLR and CAR at baseline and at predefined intervals to establish longitudinal trajectories and their association with ICH risk. Second, integrate multi-omics technologies—such as inflammatory cytokine profiling, gut microbiota metagenomics, or metabolomics in high-risk populations—to elucidate the molecular pathways through which the “inflammation–nutrition” axis contributes to cerebrovascular injury. Third, explore interventional studies to assess whether individualized management guided by NLR/CAR-based risk stratification can effectively reduce the incidence of ICH in MHD patients. Furthermore, combining cytokine profiling, metabolomics, or radiomics may help uncover the molecular mechanisms by which inflammation–nutrition imbalance contributes to cerebrovascular structural fragility.

## 5 Conclusions

In summary, this study provides the first systematic evaluation of the combined discriminative value of the NLR and CAR for intracranial hemorrhage (ICH) risk in MHD patients. The findings indicate that this dual-biomarker approach effectively reflects coexisting abnormalities in inflammation and nutritional status and offers significant incremental discriminative value. Given its simplicity and low cost, this method holds considerable promise for widespread application in clinical risk stratification and early intervention among dialysis patients, offering novel tools and strategies to help reduce ICH-related mortality.

## Data Availability

The original contributions presented in the study are included in the article/supplementary material, further inquiries can be directed to the corresponding author.
